# Effectiveness of platelet-rich plasma plus microfracture compared with microfracture alone in the treatment of knee cartilage lesions: a systematic review and meta-analysis of comparative studies

**DOI:** 10.1097/JS9.0000000000004610

**Published:** 2026-01-12

**Authors:** Abakar Mahamat Abdramane, Ahmed Abdirahman, Michael Opoku, Mingqing Fang, Yusheng Li, Xucheng Yang, Wenfeng Xiao

**Affiliations:** a Department of Orthopaedics, Xiangya Hospital, Central South University, Changsha, Hunan, China; b Xiangya School of Medicine, Central South University, Changsha, Hunan, China; c National Clinical Research Center for Geriatric Disorders, Xiangya Hospital, Central South University, Changsha, Hunan, China

**Keywords:** knee cartilage lesion, knee chondral defect, platelet-rich plasma, microfracture, meta-analysis

## Abstract

**Background:**

Platelet-rich plasma (PRP) combined with microfracture (MF) has shown promising potential in the treatment of knee cartilage lesions. Therefore, the purpose of this study was to perform a meta-analysis to critically evaluate and compare the efficacy of using PRP + MF versus MF alone for the treatment of knee cartilage lesions in terms of short- and medium-term clinical outcomes.

**Method:**

We systematically searched the PubMed, Cochrane Library, Embase, Google Scholar, and Web of Science databases for all relevant articles published from inception to July 2025. Clinical outcomes included Visual Analog Scale (VAS), International Knee Documentation Committee (IKDC), Tegner Score, Lysholm Score, and Western Ontario and McMaster Universities Osteoarthritis Index (WOMAC). An effect size with a *P*-value less than 0.05 was considered statistically significant. Statistical analysis was performed using the meta package in R (version 4.3.3).

**Results:**

A total of 11 studies, comprising 6 randomized controlled trials (RCTs) and 5 cohort studies, were included, totaling 664 patients. The main finding of this study is that the PRP + MF group demonstrated significantly improved VAS scores at the 3-, 12-, and 24-month follow-up; IKDC scores at the 24-month follow-up; Lysholm scores at the 3-, 6-, 12-, and 24-month follow-up; and Tegner scores at the 3-month follow-up compared to the MF group. However, there were no *statistically significant differences between the two groups in the WOMAC score*. Subgroup analyses showed superior Lysholm scores at 3 and 12 months and an IKDC score at 24 months in RCTs for the PRP + MF group compared to MF alone, while no significant differences were observed in VAS, Tegner, and WOMAC scores. In cohort studies, the PRP + MF group was associated with improved VAS at 3, 6, and 12 months; IKDC at 6 and 24 months, and Lysholm scores at 3 and 6 months, whereas Tegner scores showed no significant difference.

**Conclusion:**

Compared with MF alone, PRP + MF produced clinically meaningful improvement in functional outcomes and pain control. The results of the present study may provide preliminary evidence for selecting treatments for knee cartilage lesions. However, higher-quality clinical studies with more quantitative outcomes are needed to confirm these results and determine the long-term effectiveness and safety of PRP augmentation following MF.

## Introduction

Osteochondral lesions of the knee represent a significant clinical challenge, characterized by damage to both the articular cartilage and underlying subchondral bone. These lesions are particularly problematic due to the limited regenerative capacity of articular cartilage, which lacks vascular, nervous, and lymphatic supply^[1,2]^. When left untreated, such defects frequently progress to degenerative joint disease, with studies showing that 20–60% of patients undergoing knee arthroscopy exhibit chondral abnormalities^[3–5]^. The microfracture (MF) technique has emerged as a first-line surgical treatment, offering a minimally invasive approach to stimulate marrow-derived mesenchymal stem cells and promote fibrocartilage formation^[6–8]^. However, the resulting repair tissue is biomechanically inferior to native hyaline cartilage, often deteriorating within 18–36 months and providing only temporary symptomatic relief^[9,10]^.

Platelet-rich plasma (PRP) is an autologous blood-derived concentrate containing platelets at levels higher than those found in circulating blood. Produced through centrifugation, PRP has gained traction in musculoskeletal medicine due to its regenerative potential, particularly for tissues with poor intrinsic healing capacity^[11,12]^. This therapeutic approach has recently emerged as a promising adjunct to MF therapy, as it delivers a concentrated mixture of growth factors capable of enhancing chondrogenesis, inhibiting cartilage degradation, and modulating inflammatory responses^[13–18]^. Preclinical studies have demonstrated that PRP can improve the quality of repair tissue when combined with MF, showing better histological characteristics and more extensive defect filling compared to MF alone^[19,20]^.

Many clinical studies involving human participants have similarly documented that PRP augmentation may improve short-term outcomes, including reduced pain and enhanced functional recovery, and can accelerate and prolong the therapeutic effect of MF treatment^[1,21–24]^. PRP, owing to its beneficial role in the wound-healing process, is commonly employed in the treatment of tendon, cartilage, or muscle injuries. A meta-analysis conducted by Woo *et al*^[25]^ demonstrated that PRP augmentation in MF surgery for osteochondral lesions of the talus results in significantly improved final pain and functional scores compared to MF alone, indicating that PRP may enhance treatment efficacy. Regarding knee cartilage injuries, He *et al*^[24]^ reported that the combination of arthroscopic MF with PRP complexes improves clinical outcomes in the treatment of knee cartilage injuries, leading to greater pain relief, enhanced joint function, and increased activity levels compared to MF alone. Nevertheless, the literature remains divided, with some studies reporting no significant additional benefit from PRP. Manunta *et al*^[26]^ found that while patients treated with MF plus PRP had slightly better functional outcomes and faster pain resolution at 12 months, the difference compared to MF alone was not statistically significant. Mancò *et al*^[27]^ also found that although PRP combined with MF led to better short-term improvements in pain and function, no significant differences were found between the groups at 2 years. The conduct and reporting of this study adhered to the TITAN (Transparency In The reporting of Artificial INtelligence) guidelines 2025 for the ethical use of artificial intelligence in research^[28]^.

Over the past decade, the use of PRP as an adjunct to MF has been increasingly investigated to enhance the therapeutic outcomes in the management of knee cartilage lesions. Nevertheless, comparative evidence directly evaluating MF alone versus MF combined with PRP remains limited. Therefore, the objective of this study was to conduct a systematic review and meta-analysis (SRMA) with a rigorous selection of randomized controlled trials (RCTs) and cohort studies to compare and critically appraise the short- and medium-term clinical outcomes of PRP + MF versus MF alone for treatment of knee cartilage lesions. We hypothesized that the addition of PRP to MF would demonstrate superior pain relief, functional improvement (improvement in joint mobility and daily functional performance), and cartilage repair durability (long-term stability of regenerated cartilage), providing supportive evidence for clinical practice guidance.

## Method

This SRMA was conducted in compliance with the Preferred Reporting Items for Systematic Reviews and Meta-Analysis (PRISMA) guidelines^[29]^. The methodological quality of this study was assessed using the AMSTAR 2 (A Measurement Tool to Assess Systematic Reviews) tool^[30]^. A complete protocol and relevant considerations of this SRMA have been written and uploaded into the International Prospective Register of Systematic Reviews (PROSPERO).

**HIGHLIGHTS**

- First systematic review and meta-analysis comparing platelet-rich plasma (PRP) + microfracture (MF) to MF alone for the treatment of knee cartilage lesions.
- PRP combined with MF improves pain and functional outcomes.
- PRP shows as a promising biological adjunct to MF in knee cartilage restoration.

### Literature search

Two authors independently conducted a systematic search for studies reporting knee cartilage lesions or knee chondral defect cartilage treatment with PRP using PubMed, The Cochrane Library, Embase, Google Scholar, and Web of Science databases for all papers published from inception to July 2025, with a limitation to the English language. The comprehensive search strategies utilized combinations of free words and Medical Subject Headings (MeSH) terms, emphasizing key terms or phrases: (“Knee cartilage Lesion” OR “Knee Chondral defect” AND (“Platelet-Rich Plasma” OR “PRP”) AND (“Microfracture” OR “Bone marrow”). To achieve a more exhaustive compilation of significant studies, the same two authors independently performed a manual search of the primary references listed in the included studies, meticulously scrutinizing and documenting each source to optimize the acquisition of relevant information.

### Study eligibility

The initial step involved removing duplicate literature. Two authors independently screened titles and abstracts to identify eligible articles. When a study could not be excluded based on its title and abstract, both reviewers examined the full text to reach consensus on inclusion or exclusion, consulting a third author for significant discrepancies.

### Inclusion criteria

This SRMA concentrated on integrating studies that met the specified inclusion criteria: (1) comparative studies (including RCTs and cohort studies); (2) patients with focal knee cartilage lesions or chondral defects classified as OLT (Outerbridge–Lysholm–Tegner) grade I–V or Kellgren–Lawrence grade I–II, who were treated with either MF alone or MF combined with PRP; (3) studies that compared PRP plus MF with MF alone; (4) studies with a minimum follow-up of 3 months; (5) studies in which any of the following and clinical outcomes was reported: visual analogue scale (VAS) score, Tegner score, Lysholm score, International Knee Documentation Committee (IKDC) score, and Western Ontario and McMaster Universities Osteoarthritis Index (WOMAC) score.

### Exclusion criteria

The exclusion criteria were as follows: (1) non-clinical studies (such as *in vitro* or animal studies, reviews, case reports, conference, and editorials); (2) patients with diffuse osteoarthritis, rheumatoid arthritis, or systemic inflammatory joint diseases; (3) studies that have combined PRP with other biologic adjuncts (e.g., stem cells and hyaluronic acid); (4) studies on other joints (e.g., hip, ankle, and shoulder); (5) the full text of the literature is not available; (6) articles not in English language; (7) studies lacking the desired clinical outcomes; and (8) studies without relevant quantitative results.

### Data extraction

Two authors independently undertook the data extraction process from the finalized included literature, utilizing a structured literature information form. A consensus was reached with a third reviewer to resolve conflicts and disagreements. The data extraction process included the following information: (1) basic characteristics of the literature: title, first author, year of publication; (2) experimental information: study design, inclusion criteria, and exclusion criteria; (3) patient information: number of patients in each group, lesion size, OLT grade, gender ratio, age, PRP application, body mass index (BMI), and duration of follow-up; and (4) outcomes as previously mentioned.

### Quality assessment

To evaluate the risk of bias in RCTs and cohort studies, two independent authors used the Cochrane Collaboration’s risk of bias^[30]^ to assess the quality of RCTs and the Newcastle–Ottawa Scale (NOS)^[31]^ for cohort studies. Initially, the intention was to resolve disagreements through negotiation between the two authors. If this was unsuccessful, the decision was delegated to a third author. The Cochrane scale incorporates seven items: (1) random sequence generation; (2) allocation concealment; (3) blinding of participants and personnel; (4) blinding of outcome assessment; (5) incomplete outcome data; (6) selective reporting; and (7) other bias. Each item will be categorized as low risk, high risk, or unclear. NOS scale comprises three domains: (1) selection (subdivided into four parts, each section scoring up to one point); (2) comparability (a subsection, scoring a maximum of two points); and (3) outcome (segmented into two parts, each part gets a maximum of one point). Each study could attain a maximum of nine points, with scores interpreted as follows: 7–9 (high quality), 4–6 (medium quality), and 0–3 (low quality).

### Statistical analysis

All statistical analyses were conducted using the meta package in R (version 4.3.3). For continuous outcomes, such as VAS, IKDC, Lysholm, Tegner, and WOMAC scores, data were summarized as means and standard deviations, or, when unavailable, medians and quartiles. The inverse-variance method was used for pooling, and results were presented as mean differences (MDs) with 95% confidence intervals (CIs). A *P*-value less than 0.05 was considered statistically significant. We used the *I*^2^ statistic to assess heterogeneity between the studies. According to the Cochrane Handbook, a fixed-effect model was applied when the *I*^2^ statistic was less than 50%; otherwise, a random-effects model was deemed appropriate. When the authors of an article did not provide the standard deviation, it was estimated from the median, mean, and range using the method proposed by Hozo *et al*^[32]^ We performed meta-regression using follow-up time as a covariate and sensitivity analyses using the leave-one-out method to explore and minimize potential sources of heterogeneity. Subgroup analyses were conducted for sufficient available data according to the study design (RCTs and cohort studies). Funnel plots were used to detect publication bias.

## Results

### Literature selection

**Figure 1.**
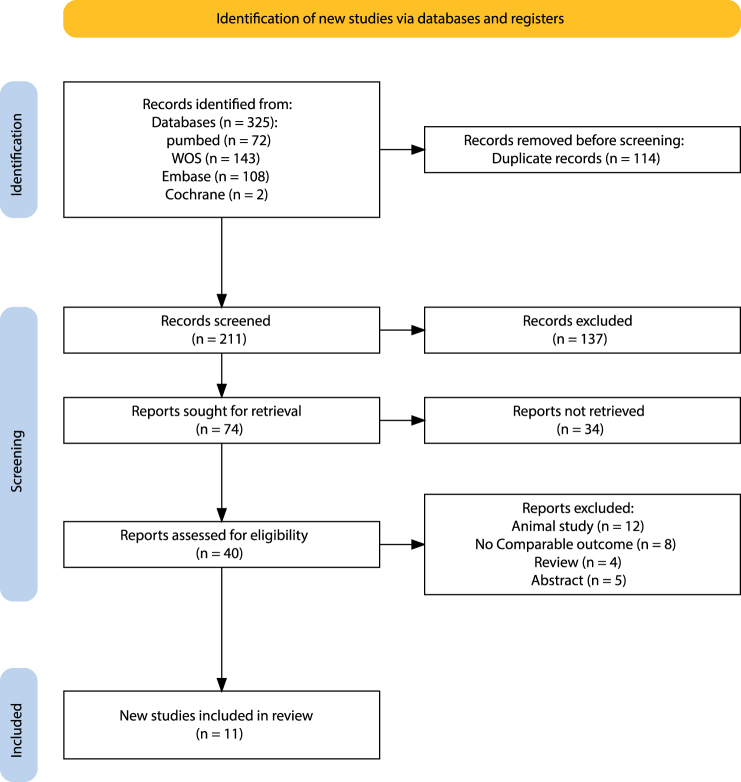
Flow chart of literature search and screening.

The systematic search strategy identified 325 potentially relevant articles across all databases queried. After removing 114 duplicate records, pre-screening was conducted by reviewing the abstract and title based on specific inclusion and exclusion criteria to identify 74 studies. After a comprehensive screening of the entire texts, 40 articles were selected for further assessment. Following the screening process, 29 unqualified articles were excluded for several reasons, including animal studies (*n* = 12), no comparable outcome (*n* = 8), review (*n* = 4), and abstract (*n* = 5). In the end, 11 studies that met the predetermined inclusion criteria were selected for inclusion in this SRMA. Figure 1 illustrates the flow chart of the systematic literature search and screening.

### Basic characteristics of the literature

The 11 included studies^[1,22–24,26,27,33–37]^ were published in English-language journals between 2013 and 2025, comprised 6 RCTs and 5 cohort studies, containing a total of 664 patients. Among these, 338 patients (50.09%) were assigned to the PRP + MF group, while 326 patients (49.06%) were assigned to the MF alone group. The included studies exhibited variability in sample size (10–65 patients) and mean patient age (32–63 years). There were 326 (49.09%) male patients and 311 (46.83%) female participants in the included studies, with one study not reporting. Patients in the included studies were followed for 3–60 months postoperatively. The MF surgical technique was executed using arthroscopy in all studies. Regarding PRP application, it was administered via distinct methods across the included studies. Three studies employed a single direct intra-articular injection under arthroscopic guidance after the MF procedure^[23,27,33]^. Additionally, six studies used a series of one to six intra-articular injections over several weeks postoperatively^[1,22,24,26,34,36]^. However, only one study did not mention the injection timing and method of PRP administration^[35]^. Various volumes of PRP were utilized at each site, as reported in all of the studies, ranging from 2 to 8 mL, whereas four studies did not provide any information about the volume of PRP used^[23,26,35,37]^. The volume of whole blood obtained from patients to prepare the PRP ranged from 8 to 200 mL, as reported in all the included studies, although only one study did not specify the volume of blood used^[26]^. Diverse types of processing machines were selected for PRP preparation, and the double-spinning process was used in three studies^[1,24,36]^. We subsequently conducted subgroup analyses of outcomes by study design (RCTs and cohort studies). The basic characteristics of the included studies, PRP preparation, and PRP application are detailed in Tables 1, 2, and 3, respectively.

**Table 1:** Basic characteristics of the included studies.

First author-year	Study design	OLT Grades (I–IV)		Lesion size		Sex (male/female)		Age (years)		Follow-up (months)	Sample size		BMI (kg/cm^2^)	
		PRP + MF	MF	PRP + MF	MF	PRP + MF	MF	PRP + MF	MF		PRP + MF	MF	PRP + MF	MF
Manuta-2013^[26]^	RCT	II–III		NR		9/11		33–55		12	10	10	NR	
Lee-2013^[33]^	RCT	III–IV		<4 cm^2^		14/10	15/10	46 (41–48)	46 (42–47)	24	24	25	27 (22–29)	28 (23–30)
Zedde-2015^[34]^	RCT	III–IV		<2 cm		16/14	18/12	32.6 ± 6.06	33.8 ± 6.7	48	30	30	24.9 ± 0.2	25.3 ± 0.3
Mancò-2016^[27]^	Prospective comparative study	III–IV		<4 cm^2^		NR		52.4		24	14	13	NR	
Papalia-2016^[35]^	Retrospective comparative study	III = 6	III = 7	4.01 ± 0.27 cm^2^ (2–6 cm^2^)		9/10	6/11	58.60 ± 6.48	58.20 ± 5.71	60	17	19	Normal weight/overweight/obesity I/obesity II:	Normal weight/overweight/obesity I/obesity II:
		IV = 11	IV = 12											
													4/5/5/1	4/8/3/0
Krakowski-2020^[23]^	RCT	IV = 25		NR		5/11	5/8	63 ± 3.8	59 ± 6.8	6	16	13	30.6 ± 2.85	31 ± 4.1
		III = 4												
Yang-2021^[22]^	RCT	NR		NR		23/17	21/18	48.95 ± 16.37	50.34 ± 15.75	3	40	39	23.42 ± 1.77	24.18 ± 1.81
Gu-2023^[1]^	Retrospective comparative study	I = 6	I = 4	NR		38/27	31/24	<40:48	<40:15	12	65	55	<24.56	<24.20
		II = 10	II = 8											
		III = 30	III = 26					≥40:17	≥40:40				≥24.9	≥24.35
		IV = 19	IV = 17											
He-2024^[24]^	Retrospective comparative study	III = 29	III = 28	NR		28/32	25/35	47.57 ± 9.552	48.02 ± 9.024	6	60	60	27.53 ± 3.18	27.92 ± 2.46
		IV = 31	IV = 32											
Mohamed-2024^[36]^	RCT	II = 18	II = 16	NR		24/26	24/26	46.62 ± 5.17	46.98 ± 5.79	12	50	50	24.58 ± 1.78	24.45 ± 1.73
		III = 21	III = 21											
		IV = 11	IV = 13											
Visoianu-2025^[37]^	Prospective comparative study	NR		NR		7/5	8/4	18-60		12	12	12	NR	

RCT, randomized controlled trial; NR, not reported; OLT, Outerbridge–Lysholm–Tegner; MF, microfracture; PRP, platelet-rich plasma; BMI, body mass index.

Data are expressed as Mean ± standard deviation or median (interquartile range).

**Table 2:** Preparation and application of platelet-rich plasma for RCTs.

Author-year	Processing machine	Whole blood volume (mL)	Anticoagulants	Spin speed (rpm)	Spin time (min)	Agent activators	Administration	Volume (mL)
Manuta-2013^[26]^	GPS system II (Biomet Biologics, Warsaw, IN, USA)	NR	Citrate dextrose solution formula – A (ACDA)	3200	15	Sodium bicarbonate (NaHCO_3_)	One week, 4 months, and 8 post-operation	NR
Lee-2013^[33]^	Magellan Autologous Platelet Separator (Medtronic Biologic Therapeutics and Diagnostics, Minneapolis, USA)	54	Sodium acid	NR	NR	No activator used	Intraoperative	6
Zedde-2015^[34]^	A centrifuge (single-spin technique)	30	NR	3200	15	NR	Seventh, fourteenth, and twenty-first day after surgery	3
Krakowski-2020^[23]^	Arthrex Angel System™	60	NR	NR	NR	Collagen	Intraoperative	NR
Yang-2021^[22]^	A centrifuge (single-spin technique)	10	NR	2000	10	Calcium chloride	Once every 7 days for a total of six cycles	2
Mohamed-2024^[36]^	A centrifuge (double-spin technique)	10	Sodium citrate	1st: 215	1st: 10	NR	Once every 3 weeks after surgery	3
				2nd: 863	2nd: 20

**Table 3:** Preparation and application of platelet-rich plasma for cohort studies.

Author-year	Processing machine	Whole blood volume (mL)	Anticoagulants	Spin speed (rpm)	Spin time (min)	Agents activators	Administration	Volume (mL)
Mancò-2016^[27]^	Triple bags (blood bag, Terumo; Penpol Ltd, Trivandrum, India)	150–200	NR	1st: 462	1st: 10	Ca gluconate	Intraoperative	6–8 (in two aliquots)
				2nd:3962	2nd: 6			
Papalia-2016^[35]^	Regen Lab THT tube platelet separator (contains a special gel)	8	NR	3100	9	NR	NR	NR
Gu-2023^[1]^	A centrifuge (double-spin technique)	20	NR	Twice: 2000 r/min		NR	once a week on three consecutive occasions	3
He-2024^[24]^	A centrifuge (double-spin technique)	20	NR	1st: 1600	1st: 15	Autologous thrombin	One Intraoperative and 14 days post-operation for 2 cycles	3
				2nd:3500	2nd: 10			
Visoianu-2025^[37]^	Arthrex ACP	16	NR	1500	5	NR	30 minutes after surgery	NR

NR, not reported; GPS, gravitational platelet separation; Ltd, Limited; ACP, autologous conditioned plasma; ACDA, anticoagulant citrate dextrose solution formula A; ™, Trademark.

### Quality assessment

The risk of bias assessment for each included study is systematically presented in Tables 4 and 5. For the RCTs, the quality assessment revealed generally low risks of bias across most domains, with some areas of concern. Two studies^[23,33]^ showed the highest methodological clarity, demonstrating a low risk across the majority of domains. However, several studies exhibited unclear risks, particularly in random sequence generation, allocation concealment, and blinding of participants and personnel. Despite these limitations, all studies maintained a low risk of attrition and reporting bias, indicating acceptable completeness of outcome data and minimal selective reporting. The quality assessment of cohort studies showed generally high methodological standards, with scores ranging from 7 to 8.

**Table 4:** Quality assessment of the randomized controlled trials.

Study-year	Random sequence generation	Allocation	Blinding of participants and personnel	Blinding of outcome assessment	Incomplete outcome data	Selective reporting	Other bias
Manuta-2013^[26]^	Low	Unclear	Unclear	Unclear	Low	Low	Unclear
Lee-2013^[33]^	Low	Unclear	Unclear	Low	Low	Low	Low
Zedde-2015^[34]^	Low	Unclear	Unclear	Unclear	Low	Low	Unclear
Krakowski-2020^[23]^	Low	Low	Low	Unclear	Low	Low	Low
Yang-2021^[22]^	Low	Unclear	Unclear	Unclear	Low	Low	Low
Mohamed-2024^[36]^	Low	Unclear	Unclear	Unclear	Low	Low	Low

Low risk indicates adequate methodological conduct and a low likelihood of bias; high risk indicates methodological flaws that may introduce bias; and unclear risk indicates insufficient information to determine the potential risk of bias.

**Table 5:** Quality assessment of the cohort studies.

Study	Selection	Comparability	Outcome	Total score
Mancò-2016^[27]^	***	**	**	7
Papalia-2016^[35]^	***	**	***	8
Gu-2023^[1]^	***	*	***	7
He-2024^[24]^	***	**	***	8
Visoianu-2025^[37]^	***	**	***	8

* = one star awarded/one score, ** = two stars awarded/two scores, and *** = three stars awarded/three scores. Scores were interpreted as follows: 7–9 (high quality), 4–6 (medium quality), and 0–3 (low quality), with each study attaining a maximum of nine points.

NR, not reported; GPS, gravitational platelet separation; Ltd, limited; ACP, autologous conditioned plasma; ACDA, anticoagulant citrate dextrose solution formula A; ™, Trademark.

### Clinical outcomes

#### VAS score

**Figure 2.**
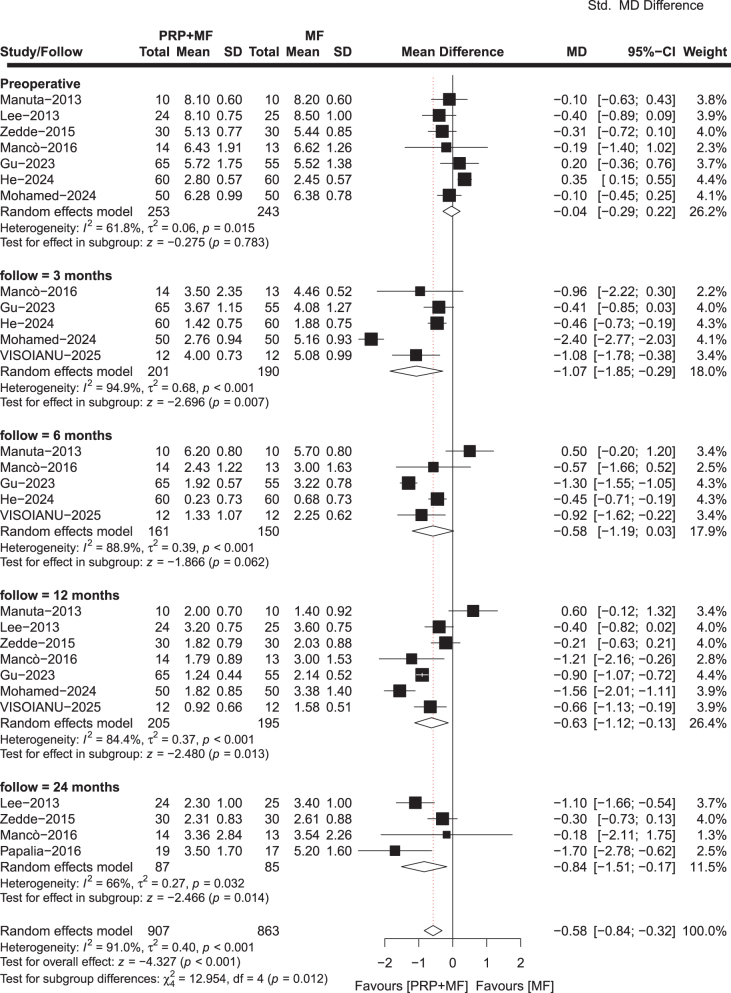
Meta-analysis results for *VAS score* between the two groups.

The VAS pain score is the most frequently employed tool for evaluating pain outcomes, and was reported in nine included studies comprising 284 patients in the PRP + MF group and 272 patients in the MF group. The final follow-up duration for these studies ranged from 3 to 24 months. Our meta-analysis results showed a statistically significant improvement in VAS pain score at 3 months (MD, −1.04; 95% CI, −1.85 to −0.23; *P* = 0.012; *I*^2^ = 91.9%), 12 months (MD, 0.80; 95% CI, −1.40 to −0.20; *P* = 0.009; *I*^2^ = 85.8%), and 24 months (MD, −0.63; 95% CI, −1.10 to −0.16; *P* = 0.008; *I*^2^ = 54.5%) in the PRP + MF group compared to the MF group, but there were no statistically significant difference at 6 months (MD, −0.71; 95% CI, −1.51 to −0.09; *P* = 0.082; *I*^2^ = 88.8%) between the two groups (Fig. 2). One study^[35]^ showed that the PRP + MF group was superior to the MF group at 5-year follow-up (*P* = 0.001). Another study^[34]^ reported that the PRP + MF group was superior to the MF group at 3 and 4 years of follow-up; however, the results were not statistically significant.

#### IKDC score

**Figure 3.**
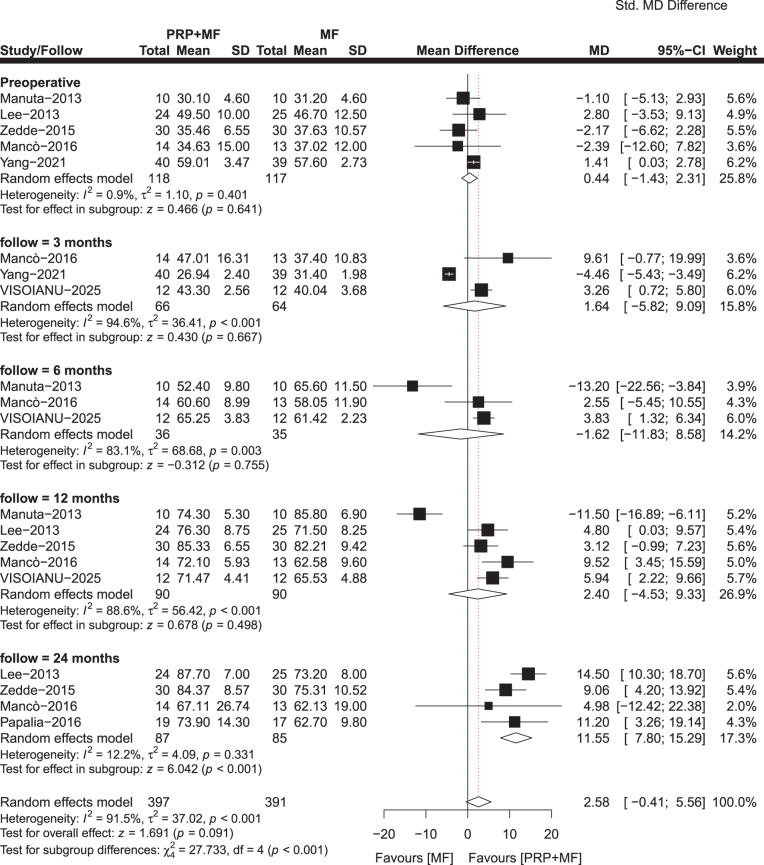
Meta-analysis results for *IKDC score* between the two groups.

The IKDC score is commonly used as a standardized outcome measure to assess the effectiveness of interventions for knee injuries, and was utilized in seven included studies involving 149 patients in the PRP + MF group and 146 patients in the MF group. The last follow-up time of these studies ranged between 3 and 24 months. The results of our meta-analysis revealed a statistically significant improvement in IKDC score at 24 months (MD, 0.99; 95% CI, 0.33–1.65; *P* = 0.003; *I*^2^ = 72.7%) favoring the patients in the PRP + MF group, but there were no significant differences at preoperative (MD, 0.07; 95% CI, −0.25 to 0.39; *P* = 0.669; *I*^2^ = 24.8%), 3 months (MD, −0.14; 95% CI, −2.02 to −1.74; *P* = 0.885; *I*^2^ = 95.9%), 6 months (MD, 0.09; 95% CI, −1.23 to 1.41; *P* = 0.891; *I*^2^ = 84.2%), and 12 months (MD, 0.35; 95% CI, −0.65 to 1.34; *P* = 0.497; *I*^2^ = 82.5%) between the two groups (Fig. 3). One study^[35]^ revealed that the PRP + MF group exhibited more favorable results than the MF group at 5-years of follow-up (*P* = 0.001). While another study^[34]^ indicated that the PRP + MF group demonstrated better outcomes than the MF group at 3 and 4 years of follow-up, the improvement was not statistically significant.

#### Lysholm score

**Figure 4.**
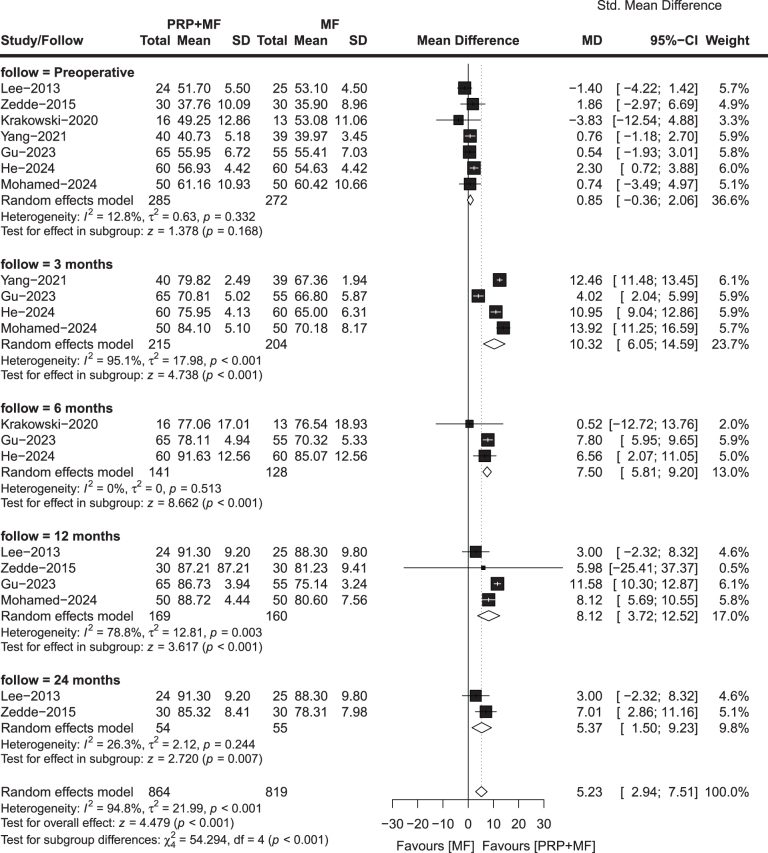
Meta-analysis results for *Lysholm score* between the two groups.

In total, seven studies comprising 285 patients in the PRP + MF group and 272 patients in the MF group reported the Lysholm score. The minimum follow-up of these studies varied from 3 to 24 months. The pooled analysis results of our study demonstrated a statistically significant improvement in Lysholm score at 3 months (MD, 10.32; 95% CI, 6.05–14.59; *P* = 0.001; *I*^2^ = 95.1%), 6 months (MD, 7.50; 95% CI, 5.81–9.20; *P* = 0.001; *I*^2^ = 0%), 12 months (MD, 8.12; 95% CI, 3.72–12.52; *P* = 0.001; *I*^2^ = 78.8%), and 24 months (MD, 5.37; 95% CI, 1.50–9.23; *P* = 0.007; *I*^2^ = 26.3%) supporting the PRP + MF group compared to the MF group (Fig. 4). At 3 and 4 years postoperatively, one study^[34]^ observed that the PRP + MF group had better results than the MF group, although the difference was not statistically significant.

#### Tegner score

**Figure 5.**
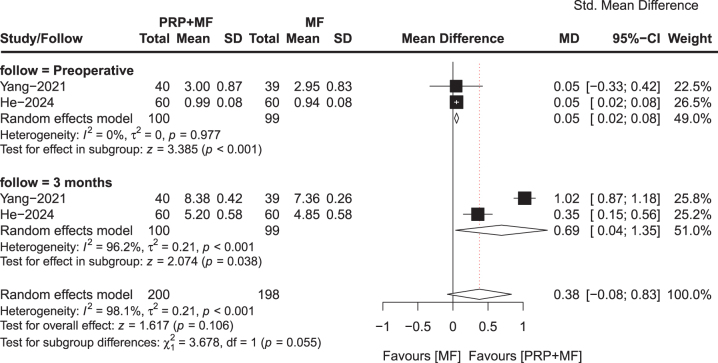
Meta-analysis results for *Tegner score* between the two groups.

Two trials involving 100 patients in the PRP + MF group and 99 patients in the MF group reported Tegner scores. The overall results of this meta-analysis showed a significant improvement in Tegner score at preoperative (MD, 0.05; 95% CI, 0.02–0.08; *P* = 0.01; *I*^2^ = 0%) and at 3 months post-operative (MD, 0.69; 95% CI, 0.04–1.35; *P* = 0.038; *I*^2^ = 96.2%) favoring the patients in the PRP + MF group compared to the MF group (Fig. 5).

#### WOMAC

**Figure 6.**
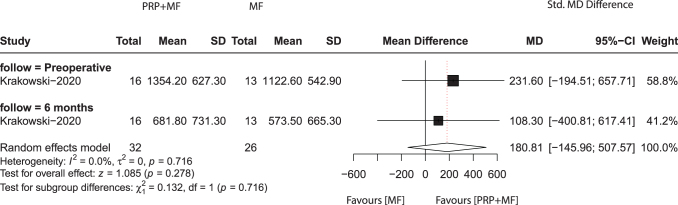
Meta-analysis results for *WOMAC score* between the two groups.

The WOMAC score is commonly used as a standardized outcome measure to assess pain, stiffness, and functional disability in patients with knee or hip osteoarthritis. It was only reported in one study, comprising 13 patients in the PRP + MF group and 16 in the MF group, who reported WOMAC scores at preoperative and 6 months. The meta-analysis results showed that there was no statistically significant difference between the two groups (MD, 180.81; 95% CI, −145.96 to 507.57; *P* = 0.716; *I*^2^ = 0.0%) (Fig. 6).

### Subgroup analysis for RCTs

**Figure 7.**
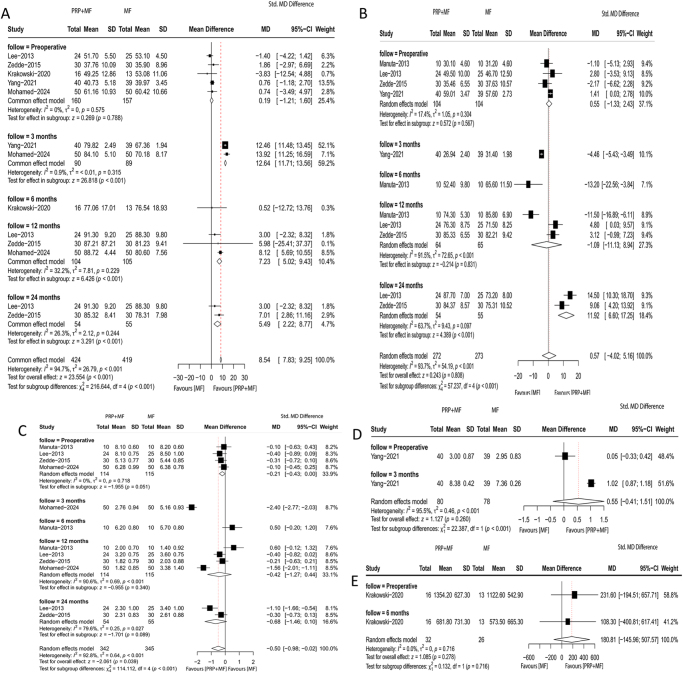
Meta-analysis results for *Lysholm score* (A), *IKDC score* (B), *VAS score* (C), *Tegner score* (D), and *WOMAC score* (E) of subgroup analysis for RCTs between the two groups.

The subgroup analysis results for RCTs showed a statistically significant differences in Lysholm score at 3 months (MD, 12.64; 95% CI, 11.71–13.56; *P* < 0.001; *I*^2^ = 0.9%), 12 months (MD, 7.23; 95% CI, 5.02–9.43; *P* = 0.001; *I*^2^ = 32.2%), and 24 months (MD, 7.71; 95% CI, 3.98–11.44; *P* = 0.001; *I*^2^ = 85.7%) (Fig. 7A); and IKDC score at 24 months (MD, 11.92; 95% CI, 6.60–17.25; *P* = 0.749; *I*^2^ = 63.7%) (Fig. 7B) favoring the PRP + MF group compared to the MF group. However, there were no significant differences in VAS score at 6 months (MD, −0.42; 95% CI, −1.27 to 0.44; *P* = 0.340; *I*^2^ = 90.6%) and 12 months (MD, −0.50; 95% CI, −1.46 to 0.10; *P* = 0.089; *I*^2^ = 79.6%) (Fig. 7C); IKDC score at 12 months (MD, −1.09; 95% CI, −11.13 to 8.94; *P* = 0.831; *I*^2^ = 91.5%) (Fig. 7B); Tegner score (MD, 0.55; 95% CI, −0.41 to 1.51; *P* = 0.260; *I*^2^ = 95.5%) (Fig. 7D); and WOMAC score (MD, 180.81; 95% CI, −145.96 to 507.57; *P* = 0.278; *I*^2^ = 0.0%) (Fig. 7E) between the groups.

### Subgroup analysis for cohort studies

**Figure 8.**
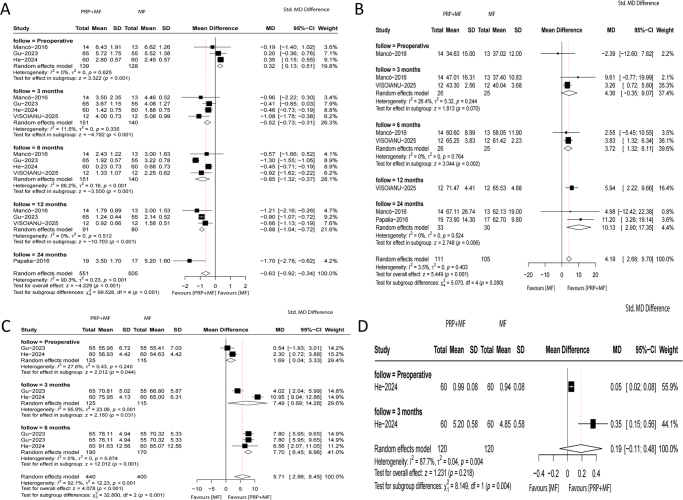
Meta-analysis results for *VAS score* (A), *IKDC score* (B), *Lysholm score* (C), and *Tegner score* (D) of subgroup analysis for cohort studies between the two groups.

The meta-analysis results of subgroup analyses for cohort studies demonstrated a statistically significant difference in VAS score at 3 months (MD, −0.56; 95% CI, −0.73 to −0.31; *P* < 0.001; *I*^2^ = 11.5%), 6 months (MD, −0.85; 95% CI, −1.32 to −0.37; *P* < 0.001; *I*^2^ = 86.2%), and 12 months (MD, −0.88; 95% CI, −1.04 to −0.72; *P* < 0.001; *I*^2^ = 0%) (Fig. 8A); IKDC score at 6 months (MD, 3.72; 95% CI, 11.32–6.11; *P* = 0.002; *I*^2^ = 0.0%) (Fig. 8B) and 24 months (MD, 10.13; 95% CI, 2.90–17.35; *P* < 0.006; *I*^2^ = 0%); and Lysholm score at 3 months (MD, 7.49; 95% CI, 0.69–14.28; *P* = 0.0031; *I*^2^ = 95.9%) and 6 months (MD, 7.70; 95% CI, 6.45–8.86; *P* < 0.001; *I*^2^ = 0%) (Fig. 8C), supporting the PRP + MF group compared to the MF group, while there were no significant differences in IKDC score at 3 months (MD, 4.36; 95% CI, −0.35 to 9.07; *P* = 0.070; *I*^2^ = 26.4%) (Fig. 8B) and Tegner score (MD, 0.19; 95% CI, −0.11 to 0.48; *P* = 0.218; *I*^2^ = 87.7%) (Fig. 8D) between the groups .

### Radiological outcomes

Only two included studies reported MRI-based outcomes. In the study by Gu *et al*^[1]^, which involved 65 patients in the PRP + MF group and 55 patients in the MF alone group, 12 months after surgery, the subchondral bone marrow edema volume and cartilage defect area of the PRP + MF group were significantly smaller than those of the MF group (*P* < 0.001), while the repaired cartilage thickness of the PRP + MF group was greater than that of the MF group (*P* < 0.001). Similarly, the study by He *et al*^[24]^, which included 60 patients in each group, used the Recht grading system and reported that after 6 months, 24 patients (40%) were classified as Grade I and 36 patients (60%) as Grade II in the PRP + MF group, indicating marked improvement compared with baseline. In contrast, in the MF alone group, 12 patients (20%) were Grade I and 48 patients (80%) were Grade II, suggesting a less pronounced improvement.

### Multivariate meta-regression

**Figure 9.**
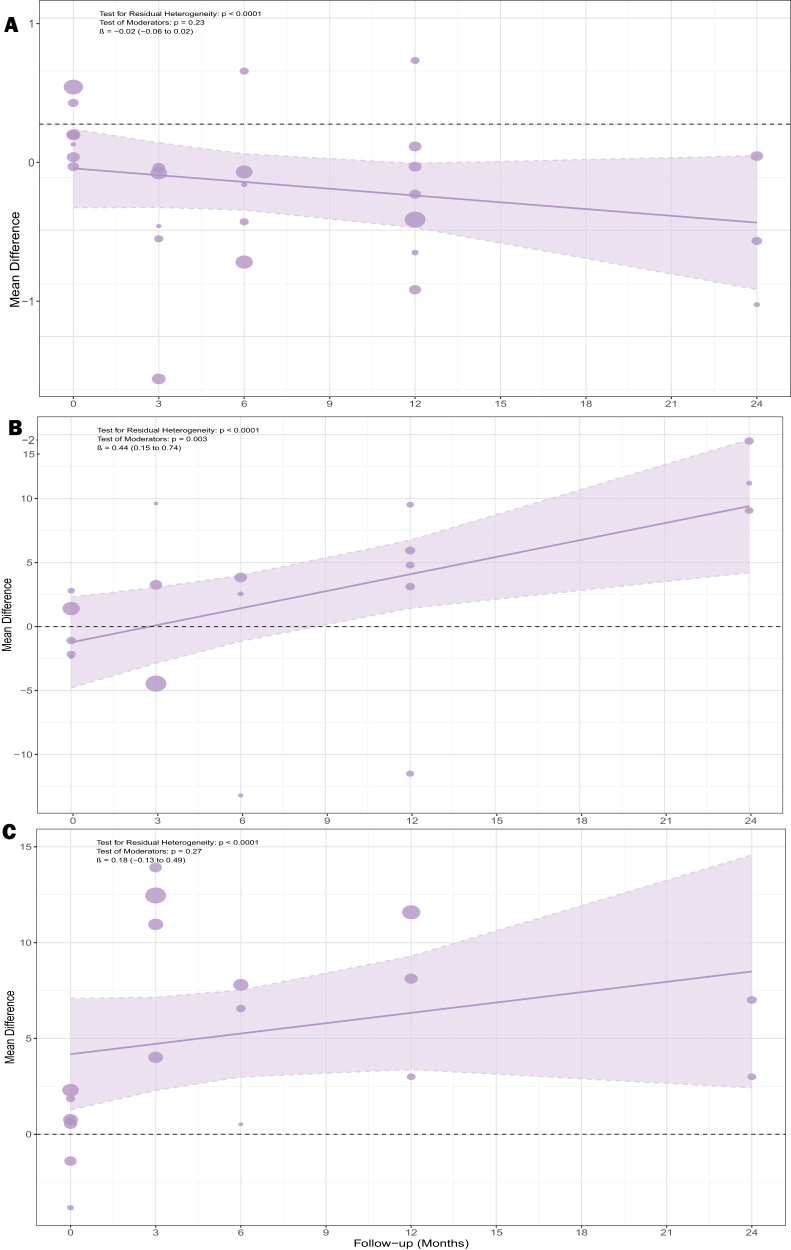
Meta-regression for *VAS score* (A), *IKDC score* (B), and *Lysholm score* (C).

We performed meta-regression for VAS score (Fig. 9A), IKDC score (Fig. 9B), and Lysholm score (Fig. 9C) using follow-up time as a covariate. The residual heterogeneity among these three measures after meta-regression remains considerable. Follow-up time had no significant effect on VAS and Lysholm scores, suggesting that the results of these two indicators did not change significantly with the extension of follow-up time. However, meta-regression showed that with extended follow-up, the difference in IKDC score between the PRP + MF group and the MF group became increasingly large (test of moderators: *P* = 0.003; β = 0.44; 95% CI, 0.15–0.74), supporting the PRP + MF group. Before conducting the meta-regression, the heterogeneity values for the VAS score (A), IKDC score (B), and Lysholm score (C) were 91.0%, 91.5%, and 94.8%, respectively. After the meta-regression, these values decreased to 89.93%, 89.23%, and 93.66%, respectively, suggesting that the included covariates accounted for only a portion of the heterogeneity, while substantial residual heterogeneity remained.

### Publication bias

**Figure 10.**
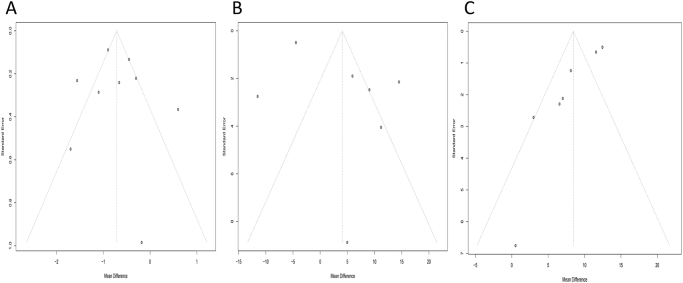
Funnel plot analysis results for *VAS score* (A), *IKDC score* (B), and *Lysholm score* (C).

We performed publication bias for VAS score (Fig. 9A), IKDC score (Fig. 9B), and Lysholm score (Fig. 9C). The funnel plots (Fig. 10) showed no apparent asymmetry, suggesting no significant publication bias among the included studies.

### Sensitivity analysis

**Figure 11.**
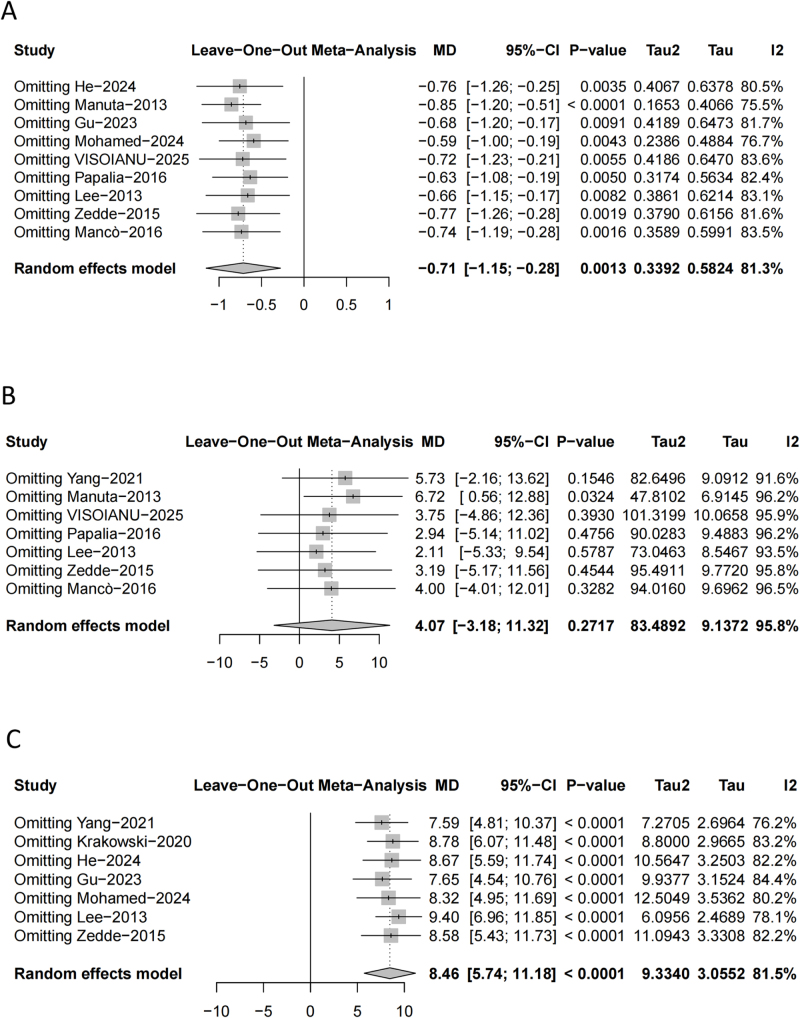
Sensitivity analysis for *VAS score* (A), *IKDC score* (B), and *Lysholm score* (C).

We conducted sensitivity analyses for VAS, IKDC, and Lysholm scores by using the leave-one-out method. For the VAS score (MD, −0.71; 95% CI, −1.15 to −0.28; *P* = 0.0013; *I*^2^ = 81.3%) (Fig. 11A), IKDC score (MD, 4.07; 95% CI, −3.18 to 11.32; *P* = 0.2717; *I*^2^ = 95.8%) (Fig. 11B), and Lysholm score (MD, 8.46; 95% CI, 5.74–11.18; *P* < 0.0001; *I*^2^ = 81.5%) (Fig. 11C), the exclusion of individual studies led to a slight increasing in effect size, while heterogeneity decreased somewhat, indicating the robustness of the results. We may attribute the subtle change in heterogeneity to differences in study design, sample size, and patient and participant allocation and blinding.

## Discussion

This SRMA, including comparative studies, aimed to evaluate and compare the efficacy of using PRP + MF versus MF alone for the treatment of knee cartilage lesions. The main finding of this study is that PRP + MF can provide better VAS scores at the 3-, 12-, and 24-month follow-up, IKDC scores at the 24-month follow-up, Lysholm scores at the 3-, 6-, 12-, and 24-month follow-up, and Tegner scores at the 3-month follow-up compared to MF alone. However, there were no statistically significant differences between the two groups in the WOMAC score. In terms of radiological outcomes, MRI showed smaller cartilage defect areas and subchondral bone marrow edema volume, greater repair thickness at 12 months, and higher Recht grades at 6 months in the PRP + MF group, indicating improved cartilage filling, maturation, and structural quality. Subgroup analyses revealed differing outcomes between RCTs and cohort studies. In RCTs, significant improvements favoring the PRP + MF group were observed in Lysholm scores at 3, 12, and 24 months and in IKDC scores at 24 months, while other outcomes, including VAS, IKDC, Tegner, and WOMAC scores, showed no significant differences. In contrast, cohort studies demonstrated significant improvements in VAS scores at 3, 6, and 12 months; IKDC scores at 6 and 24 months; and Lysholm scores at 3 and 6 months in the PRP + MF group, whereas the Tegner score showed no significant difference. Overall, these findings indicate more consistent benefits in cohort studies, potentially reflecting variations in patient characteristics, follow-up durations, or treatment protocols.

Considerable heterogeneity was observed for several outcomes, likely due to variations in patient demographics, lesion characteristics, and PRP protocols (dose, concentration, number of injections, and timing). Meta-regression using follow-up duration indicated that only the IKDC score was significantly influenced by follow-up time, suggesting that functional improvements measured by IKDC may be more dependent on longer-term assessment. Sensitivity analyses also showed that excluding individual studies led to a slight increase in effect size, while heterogeneity decreased somewhat, indicating the robustness of the results. Funnel plots did not reveal significant asymmetry for VAS, IKDC, and Lysholm scores, suggesting minimal publication bias.

MF, as an initial inexpensive and simple treatment, has been used for decades to treat small full-thickness cartilage lesions less than 2 cm^2^ in size^[38]^. Although good clinical outcomes at short-term follow-up were observed, the long-term efficacy of MF is mixed and uncertain^[39,40]^. The histological analysis revealed that the repair tissues after surgery were composed primarily of fibrous tissue/fibrocartilage, which was structurally weaker and insufficient and was susceptible to deterioration over time compared with native hyaline cartilage^[41,42]^. Several studies have begun to explore MF with various orthobiologic or scaffold augmentation to improve the quality of repair cartilage, and PRP is one of the biological agents employed in this process^[43]^.

PRP has been widely applied in regenerative medicine, as it releases numerous biologically active factors (growth factors, cytokines, and lysosomes) and adhesion proteins to promote tissue healing^[44]^. After activation, the α-granules in platelets release high concentrations of growth factors, such as platelet-derived growth factor (PDGF) and transforming growth factor β (TGF-β)^[45]^. PDGF plays a specific role in cartilage regeneration and homeostasis maintenance by promoting the proliferation of mesenchymal stem cells and inhibiting the apoptosis and inflammation of chondrocytes induced by IL-1^[46]^. Furthermore, TGF-β is active in inhibiting inflammation and stimulating chondrogenesis, and can promote mesenchymal stem cells’ chondrogenic differentiation via inter-molecular actions^[44]^. PRP is not only considered to directly induce the healing/regeneration of damaged cartilage tissue through various growth factors mentioned earlier, but also can serve as a scaffold to support cartilage regeneration and a biochemical stimulation carrier that can overcome the dedifferentiation state of chondrocytes^[45]^.

The Lysholm score is a widely used index for evaluating functional outcomes in patients with knee injuries. Therefore, it was used as an essential evaluation index in the studies included in this meta-analysis. Yang *et al*^[24]^ and Gu *et al*^[1]^ reported a positive relationship between PRP injection combined with MF and superior Lysholm scores. Moreover, these studies reported that patients in the PRP combined with MF group had higher Lysholm scores at various follow-ups than those in the control group. In contrast, Krakowski *et al*^[23]^ did not find any significant functional advantage of PRP + MF over MF alone in the final Lysholm score. The results of our meta-analysis, which pooled these findings, showed that the MF + PRP group had a superior final Lysholm score to the MF alone group at multiple time points.

In this study, the VAS score was used to assess overall pain. Many previous studies^[1,24,26,27,33–36]^ have demonstrated that patients in the MF + PRP group had lower VAS scores than the MF alone groups, which is consistent with the results of this study. The efficacy of PRP on reducing pain, as reported in the literature, may be attributed to its ability to suppress pain pathways. As suggested by Khatab *et al*^[47]^, PRP may inhibit the production of prostaglandin E2, a key mediator of inflammatory pain, and reduce the density of pain-related neuropeptides (e.g., calcitonin gene-related peptide [CGRP]) in joint tissues, thereby decreasing pain sensitization. This proposed mechanism is supported by the clinical findings of Yang *et al*^[22]^, who described that the difference in post-operative VAS scores was significantly superior in the PRP + MF group. In addition, Gu *et al*^[1]^ reported that the PRP group had a better pain level at follow-up. The results of our meta-analysis confirm that PRP augmentation provides significantly better pain control, with a notably strong effect in the early post-operative period.

The IKDC score is another instrument that provides a comprehensive evaluation of knee symptoms, function, and sports activity. This score was used as an evaluation item in several studies included in this meta-analysis. Yang *et al*^[22]^ described that the PRP + MF group had significantly better IKDC scores at 1, 2, and 3 weeks postoperatively. Our meta-analysis corroborates a functional advantage, demonstrating a significantly superior IKDC score for the PRP + MF group at the 24-month follow-up. This novel medium-term functional improvement aligns with the findings of Zedde *et al*^[34]^, who observed a slower and less pronounced deterioration of clinical results in the PRP group at 24, 36, and 48 months, suggesting that PRP augmentation may not only improve outcomes but also contribute to their greater durability compared to MF alone.

The application of PRP in various musculoskeletal-related knee joint diseases in clinical practice has been extensively studied. Costa *et al* conducted a meta-analysis to investigate the benefits and harms of intra-articular PRP injection in knee osteoarthritis. They found that PRP could provide better pain and functional outcomes, as well as comparable adverse events compared to hyaluronic acid^[48]^. Andriolo *et al* performed a meta-analysis exploring multiple non-surgical treatments for patellar tendinopathy, and the results showed that various PRP injections could provide good clinical outcomes at long-term follow-up and could therefore be considered a suitable option for the treatment of patellar tendinopathy^[49]^. In addition to the chronic inflammatory diseases mentioned earlier, PRP is also being used as an adjunct therapy for the surgical treatment of knee sports injuries. The meta-analysis conducted by Lv *et al* demonstrated that PRP injection is a safe intervention to reduce short-term pain severity after anterior cruciate ligament reconstruction. However, its efficacy in promoting functional recovery still needs to be confirmed through future well-designed and larger-scale studies^[50]^. A meta-analysis performed by Li *et al* reported that there is insufficient evidence to support an improvement in functional outcomes of meniscus repair with PRP, but that meniscus repair with adjuvant PRP showed a significantly lower failure rate and better postoperative pain control compared with meniscus repair alone^[51]^.

Numerous *in vitro* studies have confirmed the potential of PRP in promoting the repair of cartilage defects. PRP-cultured adult porcine chondrocytes exhibited stronger proteoglycan and collagen synthesis in the presence of stable cell phenotypes^[52]^. Another study showed that PRP may promote migration and stimulate chondrogenic differentiation of human subchondral progenitor cells^[53]^. In addition, PRP supports mesenchymal stem cell proliferation and chondrogenic differentiation^[54]^. Consistent with the above *in vitro* experiments, recent *in vivo* experiments have demonstrated the effectiveness of PRP in the treatment of cartilage lesions in MF surgery. Hapa *et al* found that MF plus PRP provided better cartilage healing as well as increased expression of type II collagen compared to MF alone in a rat model of chronic focal cartilage defects^[55]^. This was further confirmed in the study of Karakaplan *et al*, which demonstrated that intra-articular injection of PRP as an adjuvant of MF has a beneficial effect on the regeneration of hyaline-like cartilage in full-thickness cartilage lesions in rabbit models^[56]^. Moreover, Yasui *et al* compared single versus serial PRP injections in osteochondral lesions treated with MF and found no apparent advantage of serial PRP injections over single injections^[57]^. Platelet-rich fibrin (PRF), as a second-generation platelet concentrate product, has been explored by many researchers in recent years as a biological augmentation in MF surgery. Using a rabbit model of cartilage defects, Kinoshita *et al* found that the PRF membrane to augment healing of MF had better macroscopic and histological grades in promoting cartilage repair compared with MF alone^[58]^. The study by Balta *et al* reported that in the treatment of full-thickness chondral defects, there were no significant differences in macroscopic and histological observations between MF plus single PRF matrix injection and MF plus serial PRP injections, suggesting that the use of PRF matrix injection is recommended instead of serial PRP injections, as it is a one-time process that is done intraoperatively without the difficulty of repeating postoperatively^[59]^.

The results of these preclinical studies have given clinicians and scientific researchers great motivation to evaluate the effects of PRP biological augmentation of MF in clinical practice. Huang *et al* conducted a meta-analysis of RCTs comparing MF plus PRP with MF alone for the treatment of osteochondral lesions of talus (OLT), and found that arthroscopic MF surgery combined with PRP injection for the treatment of OLT significantly reduced pain and improved ankle function^[60]^. Therefore, we performed a meta-analysis of comparative studies for knee cartilage lesions, and the results confirmed the added benefit of PRP for MF surgery by showing a statistically significant difference for functional outcomes and pain control.

Most studies indicated that adding PRP to MF did not increase complication rates compared to MF alone, with only mild events such as swelling or stiffness reported^[1,22,24]^. However, the evidence on long-term safety is limited. Only two studies^[34,35]^ with follow-up beyond 4 years were identified, and neither reported any complications. Thus, while short-term outcomes suggest good safety, long-term complications remain uncertain and warrant further investigation. Although MF provides good function and pain relief in knee cartilage lesions, not every patient has benefited, with failure rates of 11–27% within 5 years and 6–32% at 10 years^[39]^. Poor outcome of arthroscopic MF surgery for knee cartilage injury is related to a variety of factors, including older age, larger lesion size, longer preoperative symptom duration, and previous surgery on the ipsilateral knee, particularly meniscectomy and anterior cruciate ligament reconstruction^[61]^. A systematic review conducted by Gopinatth *et al* showed limited long-term efficacy of MF surgery for the treatment of medium-size to large knee chondral defects^[62]^. Accurate staging of cartilage lesions had an important impact on treatment strategy and ultimate prognosis. However, the lesion size of talus was not consistent across the included trials, and some trials did not report the lesion size which may have had some effect on postoperative outcomes.

The administration protocol is a crucial aspect of using PRP. Different application methods of PRP, regarding concentration, site, timing, and the number of injections, may have different effects. Therefore, it seems to be of utmost importance to determine the best application method to realize the full potential of PRP. In a meta-analysis by Bensa *et al*, which compared different platelet concentrations of PRP for osteoarthritis, high platelet PRP provided better pain relief and more sustained functional improvement compared with low platelet PRP^[63]^. Another meta-analysis comparing the efficacy of multiple doses of PRP with a single dose PRP showed that two or three doses of PRP were equally effective in relieving pain and improving function in patients with osteoarthritis compared to one dose of PRP^[64]^.

The strengths of this study include a rigorous and systematic search, and supplementation of literature making the evidence we synthesized more credible; the use of comparative research designs to help minimize potential bias; and the evaluation of multiple clinical outcomes (VAS, IKDC, Lysholm, Tegner, and WOMAC) providing a comprehensive assessment of the effectiveness of combining PRP with arthroscopic MF for treatment of knee cartilage lesions. Furthermore, this study is the first to compare PRP combined with MF to MF alone in the treatment of knee cartilage lesions.

Certainly, our study also has several limitations. First, the number of studies included in this article is relatively small, and despite the total inclusion of 11 studies, the outcome indicators reported by them are quite diverse, which made quantitative synthesis difficult in meta-analysis. Secondly, substantial variations among the included studies, such as study designs, demographic data (age, sex, lesion size, and the degree of cartilage damage), and PRP protocols (dose, concentration, number of injections, and timing of administration), represent the principal sources of heterogeneity and introduced potential bias such as confounding. These variations also limited the feasibility of further subgroup analyses and may have influenced the pooled estimates. Thirdly, there are currently no studies that directly compare different PRP formulations or application methods as an adjunct to MF in the treatment of knee cartilage lesions, and the trials included in this SRMA did not provide sufficient information to conduct further exploration and analysis of the important factors that may affect the effectiveness of PRP. Finally, the results of this SRMA suggested that PRP injection, as an adjunct to MF in the treatment of knee cartilage lesions, had good short-term clinical outcomes, but did not provide any insight into the long-term clinical effect. Therefore, higher-level, longer-follow-up of clinical studies is needed to confirm the positive findings presented in the current literature.

## Conclusion

This study compared the clinical outcomes of PRP + MF over MF alone for the treatment of knee cartilage lesions, and the results showed that PRP + MF produced clinically meaningful improvement in functional outcomes and pain control compared with MF alone. The results of the present study may provide supportive evidence for the choice of treatments for knee cartilage lesions. However, higher-quality clinical studies with more quantitative outcomes are needed to confirm these results and determine the long-term effectiveness and safety of PRP augmentation following MF.

## Research registration unique identifying number (UIN)

The study protocol was registered on the PROSPERO website (CRD420251004446).
